# YTHDF1 alleviates sepsis by upregulating WWP1 to induce NLRP3 ubiquitination and inhibit caspase-1-dependent pyroptosis

**DOI:** 10.1038/s41420-022-00872-2

**Published:** 2022-05-04

**Authors:** Shuyao Zhang, Xinmin Guan, Wei Liu, Zhe Zhu, Hong Jin, Youfeng Zhu, Yun Chen, Min Zhang, Chengcheng Xu, Xu Tang, Jing Wang, Wang Cheng, Weihua Lin, Xiaoke Ma, Jianliang Chen

**Affiliations:** 1 Department of Pharmacy, Guangzhou Red Cross Hospital of Jinan University, Guangzhou, 510220 P.R. China; 2 Department of Emergency Medicine, Guangzhou Red Cross Hospital of Jinan University, Guangzhou, 510220 P.R. China; 3 Department of Endocrinology, Guangzhou Red Cross Hospital of Jinan University, Guangzhou, 510220 P.R. China; 4 Department of Respiratory Medicine, Guangzhou Red Cross Hospital of Jinan University, Guangzhou, 510220 P.R. China; 5 Department of Burns, Guangzhou Red Cross Hospital of Jinan University, Guangzhou, 510220 P.R. China; 6 Xidian University, School of Computer Science and Technology, Xi’an, 710071 P.R. China; 7 Clinical Laboratory, Cancer Hospital of Shantou University Medical College, Shantou, 515041 P.R. China

**Keywords:** Cell biology, Diseases

## Abstract

Pyroptosis is inflammation-associated caspase-1-dependent programmed cell death, which confers a crucial role in sepsis. The present study intends to investigate the regulatory network and function of the microarray-predicted YTHDF1 in caspase-1-dependent pyroptosis of sepsis. Peripheral blood of patients with sepsis was collected to determine WWP1 and YTHDF1 expression. An in vitro sepsis cell model was induced in RAW264.7 cells using lipopolysaccharide (LPS) and ATP and an in vivo septic mouse model by cecal ligation and perforation (CLP). After gain- and loss-of-function assays in vitro and in vivo, TNF-α and IL-1β levels and the cleavage of gasdermin-D (GSDMD) were detected by ELISA and Western blot assay, followed by determination of lactate dehydrogenase (LDH) activity. Immunoprecipitation and meRIP assay were performed to detect the ubiquitination of NLRP3 and the m6A modification of WWP1 mRNA. The binding of WWP1 to YTHDF1 was explored using RIP-RT-qPCR and dual luciferase gene reporter assay. It was noted that WWP1 and YTHDF1 were downregulated in clinical sepsis samples, LPS + ATP-treated RAW264.7 cells, and CLP-induced mice. The ubiquitination of NLRP3 was promoted after overexpression of WWP1. WWP1 translation could be promoted by YTHDF1. Then, WWP1 or YTHDF1 overexpression diminished LDH activity, NLRP3 inflammasomes and caspase-1-mediated cleavage of GSDMD in LPS + ATP-induced RAW264.7 cells. Overexpressed YTHDF1 restrained inflammatory response in CLP-induced mice. Collectively, the alleviatory effect of m6A reader protein YTHDF1 may be achieved through promotion of NLRP3 ubiquitination and inhibition of caspase-1-dependent pyroptosis by upregulating WWP1.

## Introduction

Sepsis is a life-threatening disorder that results from a dysfunction in host response to infection [1]. Due to its rising incidence and complexity, sepsis poses a major increasing global burden and a big challenge to intensive care clinicians and researchers [2]. Cytokine storm plays an important part in the pathogenesis of sepsis [3]. It has also been reported that the increase in peripheral blood mononuclear cells (PBMCs) is associated with the severity of sepsis [4]. Although the majority of surgical sepsis patients will have a rapid recovery, many patients will have chronic critical illness and suffer from dismal long-term outcomes [5]. Unfortunately, current therapy for sepsis is restricted to antimicrobial treatment and retaining the functions of degenerating organs [6]. In this context, it is of significance to seek novel targets for treatment of sepsis.

YTH N6-methyladenosine (m6A) RNA binding protein 1 (YTHDF1) is a reader of m6A, a type of dynamic mRNA modification regulating protein expression in multiple posttranscriptional levels [7]. Interestingly, it has been reported that m6A RNA methylation is associated with the heterogeneity of sepsis [8]. Of note, the bioinformatics prediction in the current study found WW domain containing E3 ubiquitin protein ligase 1 (WWP1) to be a differential gene in sepsis which can be recognized by YTHDF1. To our knowledge, WWP1 is identified as a HECT-type ubiquitin E3 ligase involved in a series of pathologies [9]. WWP1 can play a pivotal role in ubiquitin-proteasome pathway and is associated with many diseases including infectious diseases [10]. NOD-like receptor family pyrin domain-containing 3 (NLRP3) inflammasomes can stimulate the inflammation and induce immune cell apoptosis to exacerbate the development of sepsis [11]. Moreover, inactivation of NLRP3 inflammasomes could alleviate septic liver injury through autophagy-mediated degradation [12]. As previously reported, pyroptosis induced by the NLRP3/caspase-1 pathway could affect cognitive deficits in mice with sepsis-related encephalopathy [12]. Additionally, NLRP3-dependent caspase-1/11-GSDMD pathway was previously demonstrated to mediate pyroptosis in the hippocampus of a sepsis model [13]. Of note, the upregulation of NLRP3, cleaved caspase-1 and cleaved GSDMD that are related to caspase-1-dependent pyroptosis was found in pneumonia-induced sepsis [14]. Taking the above reports into consideration, we set out to explore whether YTHDF1 affects sepsis development through interaction with WWP1/NLRP3/caspase-1 axis.

## Results

### WWP1 was downregulated in sepsis

**Fig. 1:**
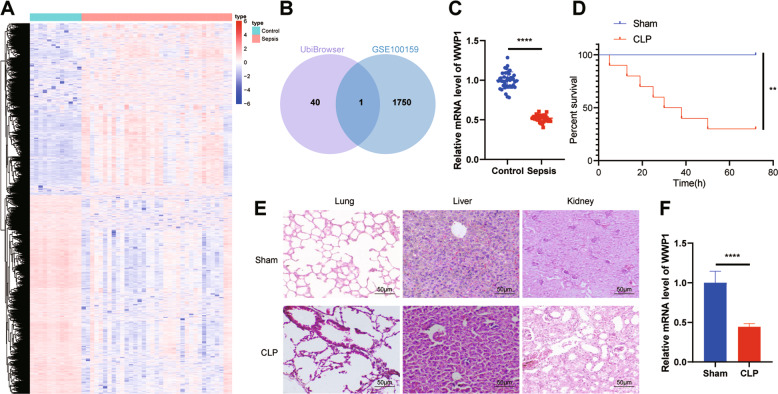
WWP1 is downregulated in sepsis. **A** The thermal map of differentially expressed genes in GSE100159 (12 normal control samples and 35 sepsis samples). **B** The intersection of UbiBrowser database and downregulated genes in GSE100159 dataset. **C** The expression of WWP1 in PBMCs collected from sepsis samples (*n* = 40) and healthy controls (*n* = 40) detected by RT-qPCR. **D** The survival rates of CLP-induced mice. **E** The pathological changes of lung, liver and kidney of CLP-induced mice observed by HE staining. **F** The expression of WWP1 in PBMCs of CLP-induced mice detected by RT-qPCR. There were ten mice in each group. **p < 0.01, ****p < 0.0001.

We predicted the E3 ubiquitin ligase of NLRP3 through UbiBrowser database (http://ubibrowser.ncpsb.org/ubibrowser/home/index) and intersected the results with the downregulated gene in the sepsis-related microarray GSE100159, whereby WWP1 was the only gene identified (Fig. 1A, B). RT-qPCR results confirmed that WWP1 was downregulated in patients with sepsis (Fig. 1C).

To further explore the role of WWP1 in the regulation of sepsis, we first established a sepsis mouse model through CLP, and analyzed the survival rate of the mice. The results revealed that the survival rate of CLP-induced septic mice was strikingly lower than that in sham-operated mice (Fig. 1D). From the results of HE staining, the CLP-induced septic mice had seriously damaged morphology of cells in lung, liver, and kidney relative to sham-operated mice, accompanied by inflammatory reaction (Fig. 1E), suggesting that the CLP mouse model was successfully established. RT-qPCR results documented that WWP1 expression in PBMCs of CLP-induced septic mice was lower than that in sham-operated mice (Fig. 1F).

These results suggest that WWP1 is poorly expressed in sepsis.

### Overexpression of WWP1 promoted NLRP3 ubiquitination to inactivate NLRP3 inflammasomes and caspase-1 mediated cleavage of GSDMD

**Fig. 2:**
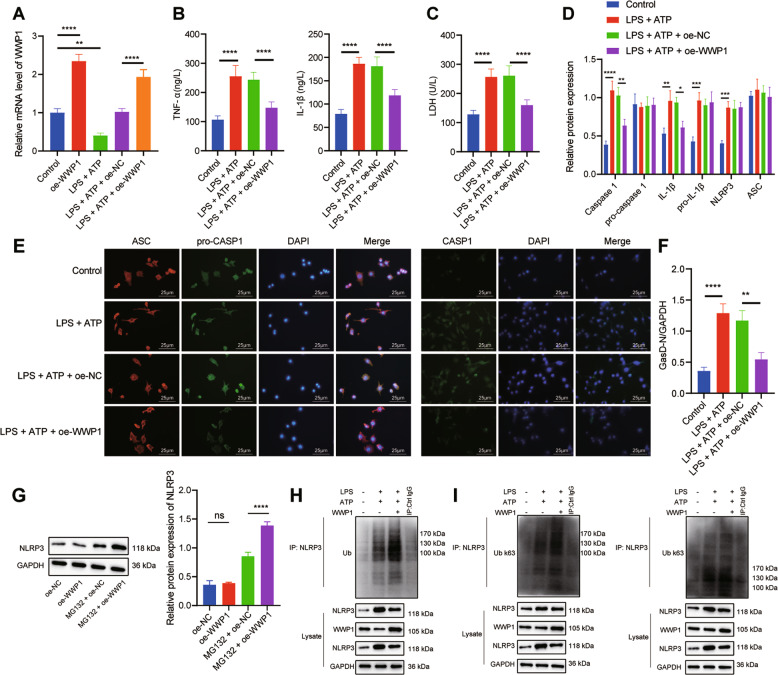
Overexpression of WWP1 promotes NLRP3 ubiquitination to inactivate NLRP3 inflammasomes and caspase-1-mediated cleavage of GSDMD. **A** The expression of WWP1 detected by RT-qPCR. **B** The expression of TNF-α and IL-1βdetected by ELISA. **C** LDH level in cells with different treatment. **D** The activation of NLRP3 inflammasomes detected by Western blot assay. **E** The co-localization of ASC with pro-caspase-1 and caspase-1, respectively in macrophages detected by immunofluorescence. **F** Protein levels of GasD-N and GasD-FL detected by Western blot assay. **G** The protein expression of NLRP3 in oe-WWP1-treated cells in response to MG132 treatment. **H** The ubiquitination of NLRP3 detected by Co-IP (WWP1: 105 kDa; NLRP3: 118 kDa). **I** K63 ubiquitination and K48 ubiquitination of NLRP3 detected by Co-IP (WWP1: 105 kDa; NLRP3: 118 kDa). *p < 0.05; **p < 0.01; ***p < 0.001; ****p < 0.0001. ‘ns’ indicates no significant difference. Cell experiments were repeated three times.

In order to further explore the molecular mechanism of WWP1 in sepsis, we treated RAW264.7 cells with LPS and ATP to construct an in vitro cell model of sepsis. WWP1 expression, as detected with RT-qPCR, was upregulated in response to oe-WWP1 treatment yet decreased following LPS + ATP treatment; in the presence of LPS + ATP, oe-WWP1 still notably increased WWP1 expression (Fig. 2A). As indicated by ELISA results, TNF-α and IL-1β levels in culture medium of cells treated with LPS + ATP had notable increases, which could be inhibited by overexpression of WWP1 (Fig. 2B). The LDH level after treatment with LPS + ATP increased significantly, while overexpression of WWP1 significantly inhibited the release of LDH (Fig. 2C). According to Western blot assay, LPS + ATP markedly activated the inflammasomes, while overexpression of WWP1 inhibited this effect (Fig. 2D). Moreover, immunofluorescence found that WWP1 overexpression led to reduced caspase-1 expression but showed no obvious influence on ASC-pro-caspase-1 spots (Fig. 2E). Western blot assay showed that LPS + ATP markedly promoted GasD-N protein expression, attenuated GasD-FL protein expression, which could be reversed by overexpression of WWP1 (Fig. 2F).

Since WWP1 restoration resulted in suppressed activation of the NLRP3 inflammasome yet unaffected NLRP3 protein expression (Fig. 2D), we speculated that WWP1 may confer a role in the degeneration of NLRP3 protein. Thus, we treated WWP1 overexpression cells with protease inhibitor MG132. Intriguingly, in the presence of MG132, an upregulation of NLRP3 expression was observed in the oe-WWP1 group relative to the oe-NC group (Fig. 2G). We further detected the level of NLRP3 ubiquitination with Co-IP. The results displayed that overexpression of WWP1 induced the polyubiquitination of NLRP3 in macrophages (Fig. 2H).

Further to explore the types of NLRP3 polyubiquitin chain, the levels of K63 and K48 ubiquitination of NLRP3 were detected by Co-IP. The results showed that overexpression of WWP1 induced K48-linked NLRP3 polyubiquitination in macrophages and had no significant effect on K63 ubiquitination of NLRP3 (Fig. 2I).

Overall, overexpression of WWP1 is able to promote NLRP3 ubiquitination while inhibiting the activation of NLRP3 inflammasomes and caspase-1-mediated cleavage of GSDMD.

### YTHDF1 promoted WWP1 expression in LPS + ATP-treated RAW264.7 cells

**Fig. 3:**
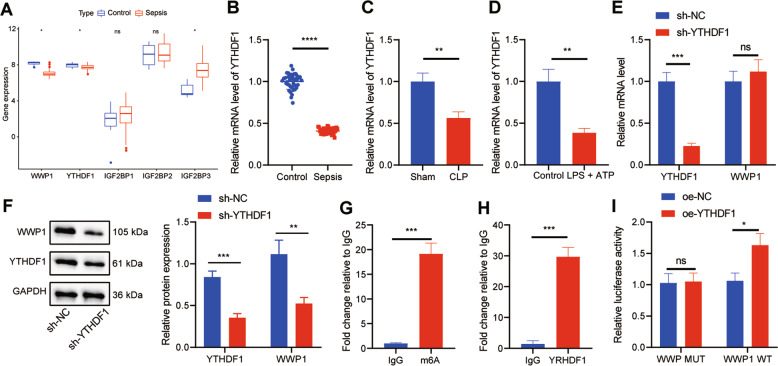
YTHDF1 facilitates WWP1 expression in LPS + ATP-treated RAW264.7 cells. **A** The expression of WWP1 and four m6A readers in sepsis-related microarray GSE00159 (measurement data were expressed as mean ± standard deviation, and paired samples *t* test was applied for comparison between the control group and the sepsis group). **B** RT-qPCR to detect the expression of YTHDF1 in PBMCs of sepsis samples (*n* = 40) and healthy controls (*n* = 40). **C** The expression of YTHDF1 in PBMCs of mice with different treatment detected by RT-qPCR. **D** The expression of YTHDF1 determined by RT-qPCR. **E** The expression of YTHDF1 and WWP1 in cells with different treatment assessed by RT-qPCR. **F** Western blot assay to measure the protein expression of YTHDF1 and WWP1 in cells with different treatment. **G** The m6A modification status of WWP1 mRNA evaluated by meRIP. H, RIP-RT-qPCR to assess the binding of WWP1 mRNA to YTHDF1 protein. **I** Dual luciferase gene reporter assay to evaluate the binding of WWP1 and YTHDF1. There were 10 mice in each group. *p < 0.05; **p < 0.01; ***p < 0.001. ****p < 0.0001. ‘ns’ indicates no significant difference. Cell experiments were repeated three times.

Furthermore, we found that there was an m6A modification site in WWP1 transcripts through the database of rmbase; therefore, we screened the m6A reader of WWP1 through the database of m6a2Target (http://m6a2target.canceromics.org/#/), which found that YTHDF1, IGF2BP1, IGF2BP2 and IGF2BP3 were potential factors to recognize the m6A modification of WWP1. Meanwhile, GSE100159 dataset manifested that YTHDF1 was downregulated and IGF2BP3 was upregulated in sepsis (Fig. 3A). It has been reported that YTHDF1 can promote the translation of target transcripts [15], indicating that YTHDF1 may alleviate sepsis by promoting WWP1 expression. We thus first used RT-qPCR to detect YTHDF1 expression in clinical samples, the results of which revealed that YTHDF1 expression was remarkably low in patients with sepsis (Fig. 3B). RT-qPCR also found that YTHDF1 expression in CLP-induced septic mice was noticeably lower than that in sham-operated mice (Fig. 3C). RT-qPCR displayed that YTHDF1 expression was markedly inhibited in RAW264.7 cells treated with LPS + ATP (Fig. 3D).

In order to further explore the effect of YTHDF1 on WWP1 expression, sh-YTHDF1 was transfected into RAW264.7 cells. Based on the results from RT-qPCR and Western blot assay, knockdown of YTHDF1 markedly downregulated the protein expression of WWP1, but had no significant effect on the RNA level of WWP1 (Fig. 3E, F). meRIP assay was implemented to assess the m6A modification status of WWP1 mRNA. The results exhibited that compared with IgG antibody, the m6A antibody notably enriched WWP1 (Fig. 3G). RIP-RT-qPCR found that compared with IgG antibody, YTHDF1 antibody significantly enriched WWP1 (Fig. 3H). Dual luciferase gene reporter assay revealed that oe-YTHDF1 resulted in a marked increase in the luciferase activity in cells after co-transfection with WWP1-WT but no difference after co-transfection with WWP1-MUT (Fig. 3I). These results suggest that YTHDF1 can upregulate WWP1 in the form of m6A in sepsis.

### Upregulation of WWP1 by YTHDF1 inhibited activation of NLRP3 inflammasomes and caspase-1-mediated cleavage of GSDMD in LPS + ATP-treated RAW264.7 cells

**Fig. 4:**
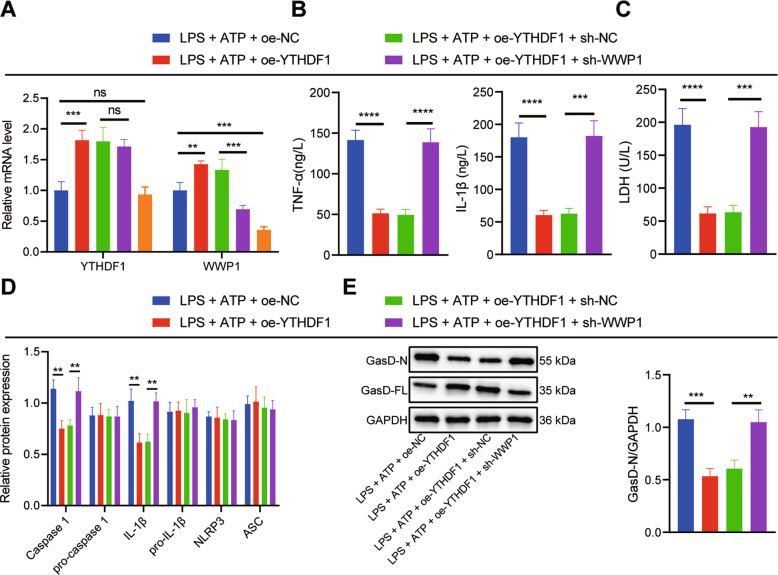
Upregulation of WWP1 by YTHDF1 inhibits activation of NLRP3 inflammasomes and caspase-1-mediated cleavage of GSDMD. **A** The expression of YTHDF1 and WWP1 in cells with different treatment assessed by RT-qPCR. **B** The expression of TNF-α and IL-1β determined by ELISA. **C** LDH level in cells with different treatment. **D** The activation of NLRP3 inflammasomes measured by Western blot assay. **E** Protein levels of GasD-N and GasD-FL detected by Western blot assay. *p < 0.05; **p < 0.01; ***p < 0.001; ****p < 0.0001. ‘ns’ indicates no significant difference. Cell experiments were repeated 3 times.

In order to further investigate the effect of YTHDF1/WWP1/NLRP3/caspase-1 axis on sepsis, RAW264.7 cells were transfected with oe-NC/oe-YTHDF1, or sh-NC/sh-WWP1. In the presence of LPS + ATP, YTHDF1 and WWP1 expression in response to oe-YTHDF1 was substantially augmented, whereas WWP1 expression was decreased yet YTHDF1 expression remained unchanged in response to sh-WWP1 treatment. In the presence of LPS + ATP + oe-YTHDF1, sh-WWP1 led to notably diminished WWP1 expression (Fig. 4A). ELISA revealed that oe-YTHDF1 brought about diminished levels of TNF-α and IL-1β in RAW264.7 cells induced by LPS + ATP, which was negated by sh-WWP1 (Fig. 4B).

Moreover, LDH level in RAW264.7 cells induced by LPS + ATP was potently lowered by overexpressing YTHDF1, which was reversed by silencing WWP1 (Fig. 4C). Based on the Western blot assay results, oe-YTHDF1 significantly inhibited the activation of NLRP3 inflammasomes in RAW264.7 cells induced by LPS + ATP, which was counteracted by further sh-WWP1 treatment (Fig. 4D). As revealed by Western blot assay, oe-YTHDF1 markedly inhibited GasD-N protein expression and augmented GasD-FL expression in RAW264.7 cells induced by LPS + ATP, which was nullified by additional sh-WWP1 treatment (Fig. 4E). Collectively, YTHDF1 can upregulate WWP1 to inhibit the activation of NLRP3 inflammasome and caspase-1-mediated cleavage of GSDMD.

### Overexpression of YTHDF1 inhibited inflammation in CLP-induced septic mice

**Fig. 5:**
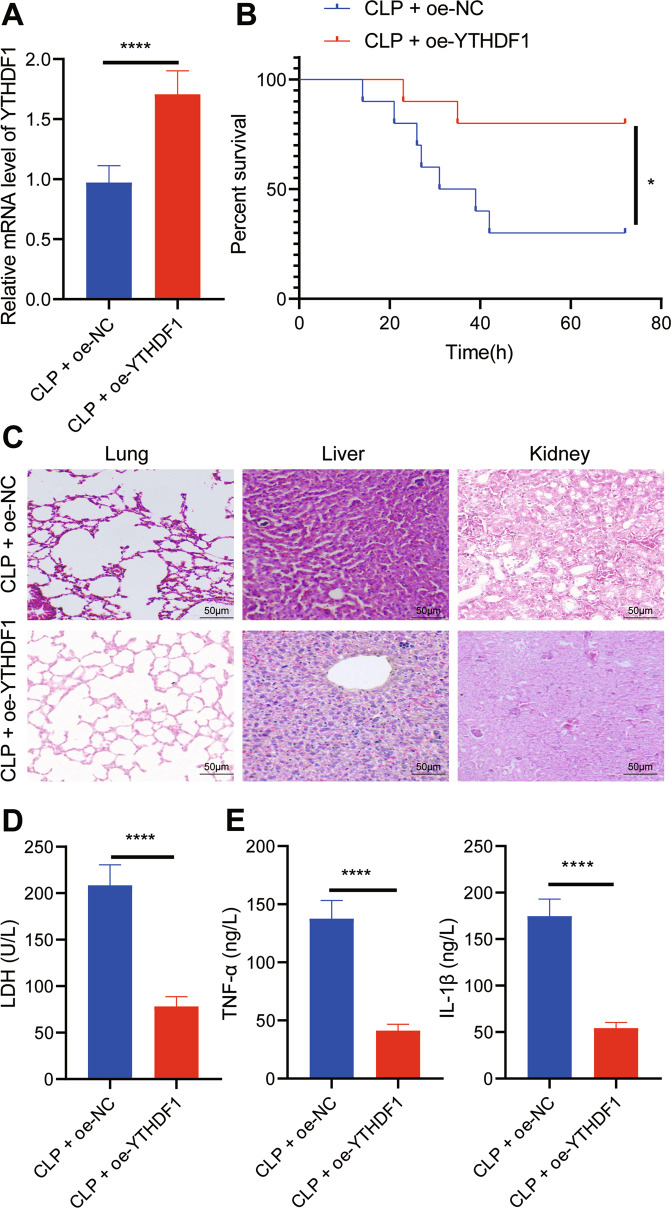
Overexpression of YTHDF1 inhibits inflammation in CLP-induced septic mice. **A** RT-qPCR to detect the expression of YTHDF1 in PBMCs of mice with different treatment. **B** The survival rates of mice with different treatment. **C** The pathological changes of lung, liver and kidney tissues of mice with different treatment observed by HE staining. **D** LDH levels in serum of mice with different treatment. **E** The expression of TNF-α and IL-1β detected by ELISA. There were 10 mice in each group. *p < 0.05; **p < 0.01; ***p < 0.001; ****p < 0.0001.

To further explore the effect of overexpression of YTHDF1 on sepsis in vivo, we overexpressed YTHDF1 in CLP-induced septic mice. From RT-qPCR results, YTHDF1 expression in PBMCs of CLP-induced septic mice treated with oe-YTHDF1 was elevated (Fig. 5A). Moreover, oe-YTHDF1 notably enhanced the survival rate of CLP-induced septic mice (Fig. 5B). The results of HE staining revealed that oe-YTHDF1 led to reduced inflammatory cell infiltration in lung, liver, and kidney tissues of CLP-induced septic mice, accompanied by restored tissue structure (Fig. 5C). In addition, oe-YTHDF1 contributed to markedly lowered LDH level in CLP-induced septic mice (Fig. 5D). ELISA demonstrated that the levels of TNF-α and IL-1β in CLP-induced septic mice treated with oe-YTHDF1 were notably restrained (Fig. 5E). These results suggest that overexpression of YTHDF1 is capable of inhibiting the inflammatory response in CLP-induced septic mice.

## Discussion

This study clarified the regulatory role of YTHDF1 in sepsis with the involvement of WWP1/NLRP3/caspase-1 axis and found that YTHDF1 could upregulate WWP1 to enhance NLRP3 ubiquitination and restrict caspase-1-dependent pyroptosis in sepsis.

In the first place, the present study revealed that WWP1 and YTHDF1 were downregulated in sepsis. It is known that YTHDF1 is the reader of m6A, which has been reported to participate in the regulation of sepsis development. As previously reported, m6A RNA methylation was accountable for the heterogeneity of sepsis [8]. Moreover, m6A modification of mRNA can exert important functions in sepsis, with m6A-cis-eQTLs showing the most obvious role in individual variation in the progression of sepsis [16]. It was revealed that upregulation of YTHDF1 in cells could contribute to reduced HIV-1 infection mainly by diminishing HIV-1 reverse transcription [17]. Notably, the role of WWP1 in inflammation and infection has also been unfolded. As previously reported, WWP1 as an E3 ligase could negatively regulate TLR4-mediated release of TNF-α and IL-6 and induce K48-linked polyubiquitination under the stimulation of LPS to regulate TRAF6 proteasomal degradation [18]. Additionally, it was revealed that WWP1 ubiquitin ligases could aid in inducing the release of HBV [19]. It is noteworthy that there is a paucity of reports regarding the relationship between YTHDF1 and WWP1. Interestingly, in the current study, it was found through rmbase-based analysis in combination with RT-qPCR and Western blot assay that YTHDF1 could promote the expression of WWP1 in sepsis.

Mechanistically, our study further demonstrated that WWP1 suppressed caspase-1-dependent pyroptosis by promoting NLRP3 ubiquitination in sepsis. Strikingly, an increasing number of studies have unfolded the involvement of NLRP3 inflammasomes in the development of sepsis and its related diseases. For instance, the inhibition of the NF-kB/NLRP3 inflammasome signaling pathway by Maf1 could lead to attenuation of sepsis-associated encephalopathy [20]. Moreover, the inactivated NLRP3 inflammasome due to melatonin could ameliorate sepsis-induced heart injury [21]. Downregulated activity of NLRP3 inflammasome in response to intravenous arginine administration alleviated acute kidney injury in a mouse model of polymicrobial sepsis [22]. Additionally, repression of the NLRP3/IL-1β axis may exert protection against endothelial relaxation dysfunction induced by sepsis [23]. It is known that proteolytic cleavage of GSDMD by caspase members including caspase-1 is a crucial step for executing pyroptosis in LPS-activated innate immune and endothelial cells and that cleaved GSDMD can stimulate NLRP3-modulated caspase-1 activation of through an intrinsic pathway [24] Caspase-1-dependent pyroptosis of PBMCs was unveiled to be able to predict the progression of sepsis in patients with severe trauma [25]. It has been reported that inactivation of NLRP3 inflammasomes and inhibition of caspase-1-mediated cleavage of GSDMD could prevent pyroptosis in LPS-induced sepsis in a mouse model, thereby alleviating the septic shock [26]. Intriguingly, the regulation on NLRP3/caspase-1 by WWP1 has been rarely reported. However, a previous study revealed that downregulation of WWP1 could activate caspase-3, thereby inhibiting growth and inducing apoptosis in hepatoma carcinoma cells [27]. Ubiquitination is one of the main mechanisms regulating the activity of NLRP3 inflammasomes, the activation of which can be inhibited by E3 ubiquitin ligase through degradation of NLRP3 [28, 29]. In the present study, we found that WWP1 could promote NLRP3 ubiquitination to repress the activation of NLRP3 inflammasomes. To conclude, the regulatory role of WWP1 in sepsis was achieved through its mediation of NLRP3 inflammasomes and caspase-1-dependent pyroptosis.

**Fig. 6:**
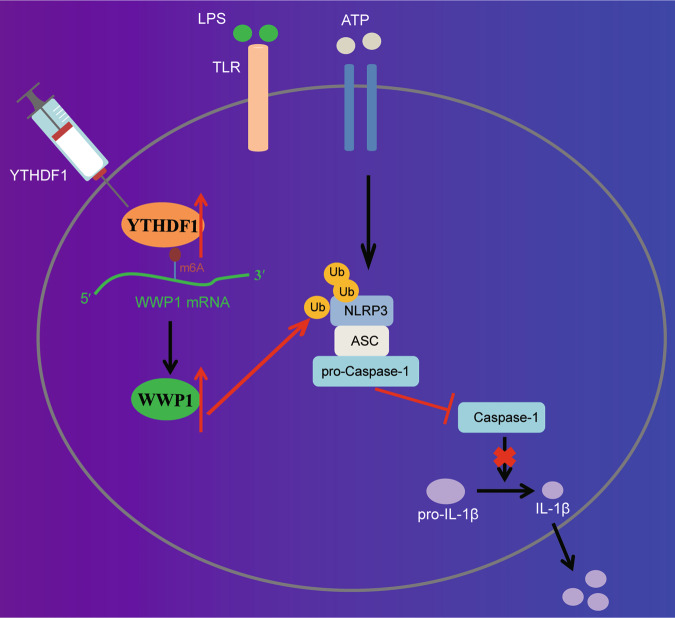
The molecular mechanism of m6A reader protein YTHDF1 in sepsis. YTHDF1 upregulates WWP1 to promote NLRP3 ubiquitination, which inhibits caspase-1-dependent pyroptosis, thereby alleviating sepsis.

Based on the results obtained in the current study, a conclusion is reached that YTHDF1 promotes NLRP3 ubiquitination by upregulating WWP1, thereby inhibiting caspase-1-dependent pyroptosis, which contributes to attenuation of sepsis (Fig. 6). This finding may provide a novel direction for diagnosis and treatment of sepsis. However, further studies are needed to validate this finding and its clinical feasibility.

## Materials and methods

### Bioinformatics methods

The sepsis-related microarray GSE100159 was downloaded from Gene Expression Omnibus database, which contained 12 normal control samples and 35 sepsis samples. R language “limma” package was adopted to identify the differentially expressed genes (DEGs) in sepsis with |log fold change (FC) | > 1 and *p* value < 0.05 as the threshold. UbiBrowser database was applied to predict E3 ubiquitin ligase of the protein, and m6A2Target database to predict mRNA m6A modifier.

### Separation of PBMCs

This study selected 40 patients with sepsis hospitalized in Cancer Hospital of Shantou University Medical College from June 2018 to June 2020, and 40 healthy individuals who underwent physical examination in the hospital at the same time period as controls. The peripheral blood samples of the study subjects (30 mL) were collected. The PBMCs were isolated from patients and healthy controls according to the instructions of kit using Ficoll-Paque-plus (GE Healthcare, Piscataway, NJ). The collected cells were stored in a refrigerator at −80 °C for subsequent experimentation.

### Cell culture

The mouse macrophage line RAW264.7 was purchased from Procell Life Science&Technology (Wuhan, China) and cultured with Dulbecco’s modified Eagle medium (DMEM) (10569044, Gibco, Carlsbad, CA) containing 10% fetal bovine serum (10099141, Gibco) and 100 U/mL penicillin and 100 μg/mL streptomycin at 37 °C with 5% CO_2_.

### Cell transfection

The RAW264.7 macrophages (4 × 10^5^ cells/well) were seeded in a 6-well plate. Upon cell confluence reaching 70–80%, cells were grouped and transfected with WWP1 overexpression plasmid (oe-WWP1), oe-YTHDF1, oe-negative control (NC), plasmids carrying short hairpin RNA targeting WWP1 (sh-WWP1), sh-YTHDF1, or sh-NC. The cell transfection was performed following the protocols of lipofectamine 2000 reagent (11668-019, Invitrogen Inc., Carlsbad, CA). Briefly, 4 μg plasmid and 10 μL lipofectamine 2000 each diluted with 250 μL serum-free medium was mixed well and added to the six-well plate after 20 min of standing. After 6-h incubation at 37 °C with 5% CO_2_ under saturated moisture, the medium containing the transfection medium was then discarded and replaced with 10% FBS-contained medium for 24–48 h for subsequent experiment. Sequences and plasmids used were all provided by GenePharma (Shanghai, China).

### Establishment of an in vitro sepsis cell model

RAW264.7 cells were treated with 500 ng/mL lipopolysaccharide (LPS) for 4 h, and then with 5 mM ATP for 30 min to develop an in vitro sepsis cell model.

### Reverse transcription-quantitative polymerase chain reaction (RT-qPCR)

The total RNA of tissue and cells was extracted using Trizol (16096020, Thermo Fisher Scientific, Rockford, IL). The complementary DNA (cDNA) was obtained via reverse transcription of the mRNA using the reverse transcription kits (RR047A, Takara, Kyoto, Japan). SYBR® Premix Ex TaqTM II kits (DRR081, Takara) were used to prepare the reaction system. The samples were subjected to RT-qPCR in a real-time fluorescence qPCR instrument (ABI 7500, ABI, Foster City, CA). Primer design is shown in Supplementary Table 1.

### Western blot

Cells were detached in RIPA lysis buffer (P0013B, Beyotime, Shanghai, China) containing 1% protease inhibitor and phosphorylase inhibitor. The total protein concentration was quantified using BCA assay kits (a53226, Thermo Fisher Scientific, Rockford, IL). After sodium dodecyl sulfate-polyacrylamide gel electrophoresis, the protein was transferred to a polyvinylidene fluoride membrane (IPVH85R, Millipore Corp., Billerica, MA). The membrane was blocked with 5% bovine serum albumin (BSA) for 1 h at room temperature, and then incubated overnight at 4 °C with primary antibodies against WWP1 (FNab09534, 1: 1000, Wuhan Fine Biotech, Wuhan, China), NLRP3 (ab263899, 1: 1000, Abcam, Cambridge, UK), YTHDF1 (ab252346, 1: 1000, Abcam), pro-caspase-1/caspase-1 (ab179515, 1: 1000, Abcam), pro-IL-1β/IL-1β (Ab234437, 1: 1000, Abcam), ASC (sc-514414, 1: 500, Santa Cruz Biotechnology, Santa Cruz, CA), GasD-FL (ab219800, 1: 1000, Abcam), GasD-N (ab215203, 1: 1000, Abcam) and glyceraldehyde-3-phosphate dehydrogenase (GAPDH) (ab181602, 1: 10000, Abcam). The membrane was then incubated with horseradish peroxidase (HRP)-labeled secondary antibody against immunoglobulin G (IgG) (ab6721, 1: 5000, Abcam) for 2 h. Enhanced chemiluminescence reagents were used to visualize the blots. ImageJ 1.48 u software (V1.48, National Institutes of Health, Bethesda, MA) was used for protein quantitative analysis through normalization of gray value of protein bands to the internal reference GAPDH.

### Coimmunoprecipitation (Co-IP)

RAW264.7 cells were lysed in protease-inhibitor cock tail (Roche Diagnostics GmbH, Mannheim, Germany)-containing cell lysis buffer (50 mM Tris HCl, pH 8.0, 150 mM NaCl, 5 mM ethylene diamine tetraacetic acid, 1 mm phenylmethylsulfonyl fluoride, 0.1% sodium dodecyl sulfate and 0.1% Triton X-100) (Sigma-Aldrich, St. Louis, MO), and the lysate was centrifuged. The supernatant was incubated with protein A/G agarose beads (Santa Cruz Biotechnology) at 4 °C for 30 min. The antibodies against NLRP3 (ab263899, 1: 30, Abcam), WWP1 (FNab09534, Wuhan Fine Biotech Co., Ltd.), ubiquitin (ab209263, 1: 30, Abcam), ubiquitin K48 (ab140601, 1: 1000, Abcam), ubiquitin K63 (ab179434, 1: 1000, Abcam), IgG (ab197767, 1: 50, Abcam) were incubated with protein A/G agarose beads in PBS containing 0.1% Triton X-100 and protein inhibitor for 10 min. Subsequently, the supernatant was incubated with antibody protein A/G agarose at 4 °C for 3 h. The precipitates were collected by centrifugation, washed, resuspended in sodium dodecyl sulfate sample buffer and analyzed by Western blot assay.

### Radioinmunoprecipitacion (RIP)

RIP kits (#17-700, Millipore Corp.) were used to detect the binding between RNA and protein. Cells with different treatment were incubated with equal volume of RIPA lysate (P0013B, Beyotime) and then centrifuged at 4 °C at 14000 rpm to obtain the supernatant. One part of the cell extract was taken out as input, and the other part was incubated with the antibody for co-precipitation. The samples and input were detached with proteinase K to extract RNA for subsequent RT-qPCR detection of target RNA. The dilution concentration of antibodies used in RIP was as follows: YTHDF1 (10 μL, FNab09572, Fine Biotech Co., Ltd.), IgG (10 μL, 10285-1-AP, Proteintech Group, Wuhan, China). The samples were mixed with the antibodies at room temperature for 30 min. IgG was taken as NC.

Methylated RNA immunoprecipitation (MeRIP) m6A kits (Millipore Corp.) were applied for evaluation of the m6A level of WWP1. According to the instructions of the kit, MeRIP was performed, followed by RNA extraction and RT-qPCR determination on WWP1 expression.

### Dual luciferase gene reporter assay

Following the instructions of Promega dual luciferase assay system, the cDNA containing full-length CDS of WWP1 was cloned into the pGL3-basic luciferase reporter gene vector (Genecreate, Wuhan, China). RAW264.7 cells were seeded in a 24-well plate. After 24 h, oe-NC/oe-YTHDF1 and pmirGLO-WWP1-wild type (WT)/pmirGLO-WWP1-mutant type (MUT) were co-transfected into RAW264.7 cells using Lipofectamine 2000 (Invitrogen Inc.). Then, 48 h after transfection, the cells were lysed and centrifuged at 12000 g for, and the supernatant was collected. The dual luciferase reporter gene assay system (E1910, Promega, Madison, WI). Each cell sample was added with 100 μL firefly luciferase working solution to detect firefly luciferase, and Renilla luciferase was detected using 100 μL Renilla luciferase working solution. The ratio between firefly luciferase and Renilla kidney luciferase was used as the relative luciferase activity.

### Immunofluorescence

The cells were fixed in 95% absolute ethanol for 15 min, incubated with 5% BSA to block non-specific staining, and then incubated with specific primary antibody against apoptosis-associated speck-like protein (ASC) (sc-514414, 1: 100, Santa Cruz Biotechnology) and caspase-1 (sc-392736, 1: 100, Santa Cruz Biotechnology) at 4 °C overnight in darkness. Next, the cells were incubated with fluorescent secondary antibody against IgG H & L (Life Technologies, Carlsbad, CA) at 37 °C for 2 h, followed by incubation with DAPI at room temperature for 15 min. Finally, the cells were observed under a confocal scanning microscope (LSM 700; Carl Zeiss MicroImaging, Inc., Thornwood, NY).

### Determination of lactate dehydrogenase (LDH) activity

The cell supernatant or mouse serum was collected, and then subjected to detection according to the instructions of LDH assay kits (MAK066-1KT, Sigma-Aldrich). The LDH activity was calculated in the samples. LDH activity (U/L) = [(measured optical density (OD) value − control OD value/standard OD value − blank OD value)] × concentration of the standard (0.2 2 μmol/mL) × 1000.

### Animal experiment

The animals used in this study were specific-pathogen-free male C57BL/6 J mice aged 8–12 weeks (purchased from Charles River, Beijing, China). The laboratory humidity was 60–65%, and the temperature was 22–25 °C. The mice were raised under 12-h light/dark cycles, with free access to food and water. The experiment was started after one week of acclimatization. The health status of mice was observed before the experiment. Ten mice were randomly assigned to each group.

The anesthetized mice were fixed in a supine (back down) position on the mouse plate. The midline incision was made on the abdominal wall of the mice to gently pull out the cecum. The feces of the upper end of the cecum were squeezed to make the end full, and the mesenteric surface blood vessels were separated. The cecal wall was punctured with a 21-g sterile needle at the midpoint between the ligation site and the top of the cecum to cause perforation. A little content in the cecum was gently squeezes out to ensure smooth perforation, and the extruded content was wiped out. After laparotomy, the cecum in sham-operated mice was gently pulled out without any treatment and then pushed back to the abdominal cavity, followed by closure of the abdominal cavity and suturing layer by layer.

The mice were grouped into sham-operated and cecal ligation and perforation (CLP)-induced septic mice (*n* = 10). The CLP-induced septic mice were subjected to CLP to establish a sepsis model, and the survival of each was monitored three days after operation. CLP-induced septic mice were further treated with oe-NC or CLP + oe-YTHDF1 (*n* = 10). CLP-induced septic mice treated with oe-NC were injected with 10 μg adenovirus-mediated oe-NC (ad-oe-NC) and those treated with oe-YTHDF1 were injected with 10 μg adenovirus-mediated oe-YTHDF1 (ad-oe-YTHDF1) 24 h before operation. The survival of mice with different treatment was monitored three days after operation. After the experiment, the mice were euthanized.

### Hematoxylin and eosin (HE) staining

After dewaxing and dehydration, sections of lung, liver and kidney tissues of mice were soaked in Harris hematoxylin for 3–8 min, hydrolyzed with 1% hydrochloric acid alcohol, and treated with 0.6% ammonia to return to blue. Sections were stained with eosin for 1–3 min, and then immersed in 95% alcohol I and II, anhydrous ethanol I and II each for 5 min, and in xylene I and II each for 5 min for dehydration and cleaning purposes. Sections were sealed with neutral gum and examined under a microscope (Nikon, Tokyo, Japan, TE200). The images were collected to analyze lung, liver, and kidney pathology.

### Enzyme-linked immunosorbent assay (ELISA)

The expression of tumor necrosis factor-α (TNF-α) (Ab208348, Abcam) and interleukin-1β (IL-1β) (ab197742, Abcam) in serum and supernatant of mouse was detected using ELISA kits. According to the instructions of the kits, 0.1 mL of cell supernatant was incubated in the reaction wells of the 96-well plate at 37 °C for 1 h (blank well was made at the same time), followed by further reaction with 0.1 mL of freshly diluted enzyme labeled antibody at 37 °C for 0.5–1 h. Subsequently, 0.1 mL of TMB substrate solution was added to each reaction well for incubation at 37 °C for 10–30 min. A total of 0.05 mL of 2 M sulfuric acid was then added to each reaction well to stop the reaction. On the Microplate Reader, the OD value of each well was measured at 450 nm and TNF-α, IL-1β and IL-6 levels were measured

### Statistical analysis

Measurement data were shown as the mean ± standard deviation from at least three independent experiments performed in triplicate. All statistical analyses were made using GraphPad Prism 5 software, with *p* < 0.05 as a level of statistical significance. The independent sample *t* test was used to analyze the data between two groups, and one-way analysis of variance (ANOVA) was used for analysis among the multiple groups. Pearson’s correlation analysis was performed to analyze the correlation between the observed indicators. *p* < 0.05 indicated that the difference was statistically significant.

## Supplementary information

Supplementary Table 1

## Data Availability

The datasets generated/analyzed during the current study are available.
