# Supplementary material for: YTHDF1 alleviates sepsis by upregulating WWP1 to induce NLRP3 ubiquitination and inhibit caspase-1-dependent pyroptosis
Source: Cell Death Discov. 2022 May 4;8:244. doi: 10.1038/s41420-022-00872-2 (PMC9068740; doi:10.1038/s41420-022-00872-2)
Supplement: Supplementary file 1 — Supplementary Table 1 [file 41420_2022_872_MOESM1_ESM.docx]

**Supplementary Table 1** Primer sequences for RT-qPCR

| Gene | Primer sequences (5’-3’) |
| --- | --- |
| WWP1 (mouse) | Forward: TCAGGGTGGGAACAGAGAAAAG |
|  | Reverse: GCAATTGATTCCGCTGAGAC |
| WWP1 (human) | Forward: TTGCTGAGCTCATGGGAAGT |
|  | Reverse: TGGTGGTAGATCCAAGCGAT |
| YTHDF1 (mouse) | Forward: ACAGTTACCCCTCGATGAGTG |
|  | Reverse: GGTAGTGAGATACGGGATGGGA |
| YTHDF1 (human) | Forward: TACAAGCACACAACCTCCA |
|  | Reverse: GTTTCGACTCTGCCGTTC |
| GAPDH (mouse) | Forward: AGGTCGGTGTGAACGGATTTG |
|  | Reverse: TGTAGACCATGTAGTTGAGGTCA |
| GAPDH (human) | Forward: AGAAGGCTGGGGCTCATTTG |
|  | Reverse: GCAGGAGGCATTGCTGATGAT |

Note: WWP1, WW domain-containing protein 1; YTHDF1, YTH N6-methyladenosine (m6A) RNA binding protein 1; GAPDH, glyceraldehyde-3-phosphate dehydrogenase; RT-qPCR, reverse transcription-quantitative polymerase chain reaction.
